# Explaining the Sex Effect on Survival in Cystic Fibrosis: a Joint Modeling Study of UK Registry Data

**DOI:** 10.1097/EDE.0000000000001248

**Published:** 2020-08-06

**Authors:** David Taylor-Robinson, Daniela K. Schlüter, Peter J. Diggle, Jessica K. Barrett

**Affiliations:** From the a Department of Public Health and Policy, University of Liverpool, Liverpool, United Kingdom; b CHICAS, Lancaster Medical School, Lancaster University, Lancaster, United Kingdom; c Department of Public Health and Primary Care, University of Cambridge, United Kingdom; d MRC Biostatistics Unit, University of Cambridge, United Kingdom.

**Keywords:** Cystic fibrosis, Joint-modeling, Registry

## Abstract

Male sex is associated with better lung function and survival in people with cystic fibrosis but it is unclear whether the survival benefit is solely due to the sex-effect on lung function.

**Methods:**

This study analyzes data between 1996 and 2015 from the longitudinal registry study of the UK Cystic Fibrosis Registry. We jointly analyze repeated measurements and time-to-event outcomes to assess how much of the sex effect on lung function also explains survival. These novel methods allow examination of association between percent of forced expiratory volume in 1 second (%FEV1) and covariates such as sex and genotype, and survival, in the same modeling framework. We estimate the probability of surviving one more year with a probit model.

**Results:**

The dataset includes 81,129 lung function measurements of %FEV1 on 9,741 patients seen between 1996 and 2015 and captures 1,543 deaths. Males compared with females experienced a more gradual decline in %FEV1 (difference 0.11 per year 95% confidence interval [CI] = 0.08, 0.14). After adjusting for confounders, both overall level of %FEV1 and %FEV1 rate of change are associated with the concurrent hazard for death. There was evidence of a male survival advantage (probit coefficient 0.15; 95% CI = 0.10, 0.19) which changed little after adjustment for %FEV1 using conventional approaches but was attenuated by 37% on adjustment for %FEV1 level and slope in the joint model (0.09; 95% CI = 0.06, 0.12).

**Conclusions:**

We estimate that about 37% of the association of sex on survival in cystic fibrosis is mediated through lung function.

## Abstract 2

Supplemental Digital Content is available in the text.

Male sex has been identified as a positive prognostic factor in cystic fibrosis.^1–3^ An apparent effect of sex on morbidity and mortality in cystic fibrosis, with males having better outcomes, has been a common finding in large epidemiologic studies, first suggested in US centers,^4^ and then confirmed in US population-level registry studies.^5,6^ There have been similar findings in the UK,^7^ with Barr et al in the UK^8^ suggesting that despite overall improved survival in the 21st century, females continue to be more likely to die below the median age of death compared with males, a pattern that has persisted since the 1960s. There has been recent debate about the sex gap, suggesting that this may be narrowing over time as a result of improving treatment.^9,10^ In terms of use of health services in cystic fibrosis, a large study in Canada further demonstrated decreased risk of hospitalization in males.^11^ Our previous study of the UK Cystic Fibrosis Registry shows that the sex difference in cystic fibrosis outcomes is clearly apparent in the UK cystic fibrosis population^12^ and this is also shown in a recent survival analysis using UK data.^13,14^ The cause of the sex gap in survival remains unclear. Lung function, as measured by the percent predicted forced expiratory volume in 1 second (%FEV1) is commonly used as a measure of disease severity in cystic fibrosis, and has been shown to be related to survival. One explanation for the sex gap in cystic fibrosis survival is that this is explained by worse lung function in females. For instance, some studies suggest that females may be more likely to become colonized with *Pseudomonas aeruginosa* leading to lung damage at an earlier age,^15^ and this may be related to the effect of estrogen.^16^ A recent review of the sex gap in cystic fibrosis has suggested that the finding of lower female survival is evident in most studies, and that evidence to suggest closure of the gap in recent cohorts is less convincing than the data supporting its continued existence. The review does suggest that in cohorts of adults with late diagnosis, and conditional on survival to age 40 years, the sex gap appears to narrow or even be reversed.^3^

In this study, we aim to quantify how aspects of an individual cystic fibrosis patient’s longitudinal profile of lung function are related to their survival prognosis; and to decompose the impact of sex on these joint outcomes. Joint modeling approaches are potentially of great utility in the context of studying outcomes in cystic fibrosis patients.^17^ Survival is of central interest and analyses often seek to adjust for lung function as a time-varying covariate, which we know is measured imprecisely with clinically significant measurement error. Furthermore, the dynamics of lung function decline are also of interest, but there is potentially informative drop-out due to the direct link between lung function and survival prognosis. Together, these properties of cystic fibrosis data (measurement error and dropout) mean that separate analysis of repeated measurement and survival outcomes is potentially inefficient, because it does not exploit the dependence between the repeated measurement process and the hazard for survival, and leads to biased estimation of the association between the two because it ignores measurement error.^18^ Joint modeling of lung function and survival offers an approach to address all of these issues.

Joint modeling has been applied to cystic fibrosis data in a few previous studies. The first of these was a center-level study by Schluchter and colleagues that modeled longitudinal FEV1 and survival simultaneously for a cohort of delF508 homozygous patients, but this study did not explore the sex effect.^17^ Subsequently, cystic fibrosis data have been used to develop methods for joint modeling, including an approach that we previously developed.^19^ In this study, we apply this novel approach for the joint modeling of lung function and survival and contrast this to commonly used approaches to adjusting for time-varying covariates in survival analyses. We use the joint model to test the hypothesis that the survival advantage for males is explained by the effect of sex on lung function.

## METHODS

We undertook a longitudinal retrospective cohort study of individuals in the UK Cystic Fibrosis Registry, which records longitudinal health data on all people with cystic fibrosis in England, Wales, Scotland, and Northern Ireland. All UK cystic fibrosis centers and clinics routinely collect data in a standardized fashion. When patients with cystic fibrosis attend a new center in the UK, they or their parents consent to collection and storage of information on the patient’s health and treatment in the Cystic Fibrosis Registry.^20^ In the UK, cystic fibrosis patients are seen in the outpatient clinic for a comprehensive annual review, including evaluation of clinical status and pulmonary function. The UK Cystic Fibrosis Registry is supported and coordinated by the UK Cystic fibrosis Trust. In the UK, the Registry is estimated to capture almost all of the cystic fibrosis population; any consenting patients attending National Health Service clinics will have annual data routinely collected into the database, 89% of whom have a “complete” dataset capturing key clinical parameters over time. The registry is carefully managed and curated to a high level of data quality, and is therefore ideally suited to the study of cystic fibrosis outcomes.^20^

### Primary Outcome and Covariates

**FIGURE 1.**
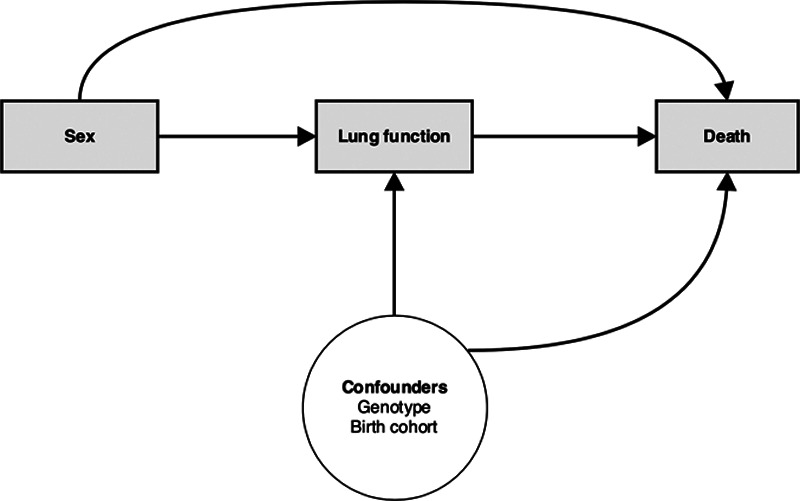
Directed acyclic graph for the effect of sex on key outcomes in cystic fibrosis. We aim to use a joint model to test the hypothesis that there is a direct effect of sex on survival.

Our directed acyclic graph which informed the analysis, identifying variables in the minimally sufficient set of adjustments, is shown in Figure 1. The primary outcomes were %FEV1, as per other studies that have explored the cystic fibrosis sex gap in outcomes,^5^ and survival. Pulmonary function tests were measured annually at the review visit, and performed according to international recommendations, measuring forced expiratory volume in 1 second, expressed as a percentage of predicted values for sex, age, height, and ethnicity using the global lung function initiative reference equations.^21^ We restricted the analysis to white patients under the age of 40 at last follow-up, with at least one lung function measurement between the start of 1996 and the end of 2015. We chose to apply an upper age limit to the analysis since the female sex gap has been shown to be present up to this point; and we have previously shown that random intercept and slope models make unrealistic assumptions when applied over long periods.^22^ About 97% of people with cystic fibrosis in the UK are white. Non-white patients tend to have worse outcomes, but the numbers are small in the UK, restricting power to demonstrate subgroup effects.^12^

The primary exposure of interest was male sex. The time metameter was patient age at clinic visit at which the %FEV1 measure was taken. Other covariates in the analysis were genotype coded as the number of delta F508 alleles (0, 1, or 2) and dichotomized into 2 F508 alleles versus 0 or 1 alleles or not typed, and birth year which was treated as a continuous covariate and centered at the mean value (approximately 1986) to capture cohort effects.

### Statistical Analysis

Full details of the joint modeling approach are provided in the supplementary eAppendix 1; http://links.lww.com/EDE/B714. We applied the method developed by Barrett et al which allows exact likelihood inference for a wide range of random-effect specifications, and the code for fitting this model is available via the link (https://github.com/Jessbarrett/CysticFibrosisJM). Repeated %FEV1 measures on individuals are correlated, and this must be accommodated to obtain valid inferences. Furthermore, lung function is related to survival. Thus, repeated measurements of %FEV1 and survival were modeled jointly using shared random effects to account for the interdependence of the two processes.^19^ The submodel for %FEV1 adjusted for the patient’s age at measurement, birth year, sex, and number of F508 alleles. Exploratory plots of the data are shown in eAppendix2; http://links.lww.com/EDE/B714. Informed by these, we approximated time-trends with a quadratic time function, to accommodate nonlinear change over time.^22^ Interactions were included between the linear time variables in age and birth year and all other covariates. The birth year was included to account for cohort effects and a survivor effect arising from left truncation of the data at the start of follow-up. The intercept (level of %FEV1 at age five) and age effect (annual linear change in %FEV1) were treated as normally distributed, correlated random effects to allow for individual intercepts and slopes. The random effects in the longitudinal model capture other sources of unmeasured heterogeneity not captured by the fixed effects.

Subjects entered the cohort at different ages so that patients contributed person-years to the analysis only at ages corresponding to their actual ages during the study period.

The probability of surviving one year was modeled using a probit link function. The survival submodel was adjusted for age halfway through the year, birth year, sex, and F508 alleles (dichotomized as previously). In addition, the survival submodel depended on a patient’s %FEV1 value halfway through the year and their %FEV1 rate of change, as estimated by the longitudinal %FEV1 model including the random effects. The time-to-event submodel is specified on a discrete-time scale modeling the conditional probability of surviving one year given that you have survived to the start of the year, whereby a positive coefficient means that an increase in the predictor leads to an increase in the predicted probability of survival. The effect of %FEV1 level and %FEV1 rate of change on survival were also expressed as hazard ratios (see the statistical eAppendix1; http://links.lww.com/EDE/B714 for details of estimating hazard ratios from our survival model). Note that for the hazard ratios, the direction of effect is reversed, i.e. a hazard ratio >1 means that an increase in the predictor gives a decreased probability of survival.

We assess whether there is a direct association between sex and survival according to the directed acyclic graph as shown in Figure 1, after accounting for lung function. Our coefficient of interest in the joint model is the sex effect in the survival submodel, after adjustment for %FEV1 rate of change and overall level of %FEV1 (for full algebraic details see eAppendix1; http://links.lww.com/EDE/B714). To demonstrate the utility of using a joint modeling approach to test our hypothesis, we assessed how the association between sex and survival changed depending on the modeling approach. Once we fitted the joint model to the data, we compared the association between sex and survival with that in a standard probit survival model, first without any adjustment for %FEV1, and then with %FEV1 added as a baseline time-invariant covariate (i.e. %FEV1 at first visit), and finally as a time-varying covariate. We estimated all model parameters by maximum likelihood and used generalized likelihood ratio statistics to compare nested models, and Wald statistics to test hypotheses about model parameters. We plotted residual diagnostics for the longitudinal and survival submodels, and an empirical variogram to check our model fit.

### Ethics

National Health Service research ethics approval (Huntingdon Research Ethics Committee 07/Q0104/2) has been granted for the collection of data into the UK database. The cystic fibrosis Trust database committee approved the use of anonymized data in this study.

## RESULTS

### Population Characteristics

The dataset contained 81,129 lung function measures on 9,741 patients between 1996 and 2015 in the UK and captured 1,543 deaths. The median number of %FEV1 measures per person was 8 (range 1–25). About 87% of individuals had three or more follow-up measures with a total of 96,598 person-years of follow-up. The baseline characteristics of the population, stratified by sex, are shown in Table 1.

**Table 1.** Characteristics of Study Population by Sex: UK Cystic Fibrosis Registry

	Female	Male	All
N (%)	4605 (47)	5136 (53)	9741
Observation, n (%)	37849 (47)	43280 (53)	81129
Observations per patient, mean (SD)	8.2 (4.7)	8.4 (4.8)	8.3 (4.8)
Deaths, n (%)	813 (53)	730 (47)	1543
Genotype			
No. delta 508: 2, n (%)	2361 (51)	2752 (54)	5113 (53)
No. delta 508: 1, n (%)	1718 (37)	1782 (354)	3500 (36)
No. delta 508: 0, n (%)	327 (7.1)	354 (6.9)	681 (7.0)
Missing	199 (4.3)	248 (4.8)	447 (4.6)
Birth cohort, n (%)			
<1960	3 (0.1)	4 (0.1)	7 (0.1)
1960–1964	68 (1.5)	115 (2.2)	183 (1.9)
1965–1969	141 (3.1)	199 (3.9)	340 (3.5)
1970–1974	257 (5.6)	335 (6.5)	592 (6.1)
1975–1979	365 (7.9)	473 (9.2)	838 (8.6)
1980-1984	587 (13)	686 (13)	1273 (13)
1985–1989	668 (15)	772 (15)	1440 (15)
1990–1994	722 (16)	749 (15)	1471 (15)
>1995	1794 (39)	1803 (35)	3597 (37)
Age at entry, yrs, mean (SD)	19.2 (9.0)	20.0 (9.4)	19.6 (9.2)
Age at diagnosis, yrs, mean (SD) (missing, n = 118)	3.0 (6.5)	3.0 (6.8)	3.0 (6.7)

### Associations of Covariates with Lung Function Trajectory

We explored the effect of covariates on %FEV1. Results from the %FEV1 submodel are shown in Table 2. Random effect parameter estimates are reported in eAppendix2; http://links.lww.com/EDE/B714. There was a borderline difference between males and females in the level of %FEV1 at age 5 with the difference in intercept of 0.90 (95% CI = 0.01, 1.80). %FEV_1_ initially declined at a rate of −1.52 (95% CI = −1.58, −1.45) percentage points per year for females. Males experienced a more gradual decline in %FEV1 (difference 0.11 per year 95% CI= 0.08, 0.14). Lung function declined at a faster rate for patients with 2 F508 alleles compared with those with 0 or 1 F508 alleles (−0.35 percentage points per year 95% CI = −0.45, −0.25).

**Table 2.** Joint Model Results for the %FEV1 Submodel. Fixed Effects Estimates of Association of Covariates on Forced Expiratory Volume in 1 second as a Percentage of Predicted (%FEV1)

	Estimate (95% CI)
Intercept at age 5 years	88 (87, 89)
Age^a^	−1.5 (−1.6, −1.4)
Age squared	0.013 (0.011, 0.015)
Birth year	0.22 (0.15, 0.29)
Male	0.90 (0.01, 1.8)
F508 alleles: 2 vs 0, 1 or not typed	−0.080 (−1.17, 1.01)
Age × male	0.11 (0.08, 0.14)
Age × F508 alleles: 2 vs 0, 1 or not typed	−0.35 (−0.45, −−0.25)
Birth year × male	0.059 (−0.027, 0.15)
Birth year × F508 alleles: 2 vs 0, 1 or not typed	−0.0040 (−0.067, 0.059)

a Age term corresponds to %FEV1 slope. The age × male term represents the age by sex interaction, i.e. the difference in slope for males compared with females

%FEV1 indicates percent forced expiratory volume.

### Associations of Covariates with Survival

**FIGURE 2.**
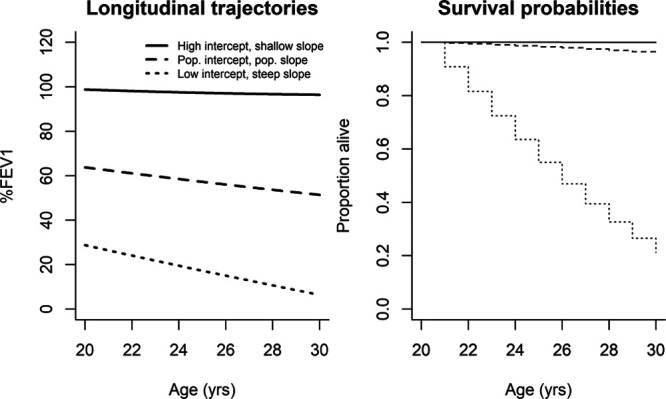
Estimated longitudinal trajectories and survival curves for 20-year-old males with varying intercepts and slopes, born in 1980, with 2 F508 alleles.

Table 3 shows the results from the survival models. The joint model (column 1) shows that higher overall levels of %FEV1 and a more gradual %FEV1 decline were associated with improved survival. Figure 2 provides a visual illustration of the relationship between lung function and survival for individuals selected from the population with different %FEV1 trajectories (different random effects). For example, for 20-year old males born in 1980 with two F508 alleles and population-average %FEV1 level and %FEV1 decline, a 10-unit lower level of %FEV1 is associated with an increase in the concurrent hazard of death (HR 2.26 CI = 2.13, 2.41). Furthermore, those with a one-unit-per-year decline compared with no decline in %FEV1 have over a three-fold higher hazard of death for every year that passes (HR 3.67 CI = 3.31, 4.07).

**Table 3.** Parameter Estimates (95% CI) From the Probit Survival Models. A Positive Coefficient Means that an Increase in the Predictor Leads to an Increase in the Predicted Probability of Survival

	Joint model	No %FEV1	Baseline %FEV1	Time-varying %FEV1
Intercept	1.6 (1.5, 1.7)	2.4 (2.3, 2.4)	1.3 (1.1, 1.4)	0.37 (0.23, 0.50)
%FEV1 (per 10 units)	0.28 (0.26, 0.29)		0.18 (0.17, 0.19)	0.32 (0.31, 0.33)
%FEV1 slope	0.45 (0.43, 0.48)			
Age-5 (per 10 yrs)	−0.042 (−−0.059, −0.026)	−0.046 (−0.089, −0.002)	−0.12 (−0.17, −0.08)	0.059 (0.007, 0.111)
Birth year (per 10 yrs)	0.26 (0.22, 0.30)	0.16 (0.12, 0.21)	−0.073 (−0.120, −0.025)	0.039 (−0.011,0.090)
Male	0.092 (0.062, 0.122)	0.15 (0.10, 0.19)	0.13 (0.09, 0.18)	0.14 (0.09, 0.19)
F508 alleles: 2 vs 0, 1 or not typed	0.14 (0.03, 0.25)	−0.13 (−0.17, −0.09)	−0.10 (−0.14, −0.06)	−0.042 (−0.089, 0.006)

%FEV1 indicates percent forced expiratory volume.

**FIGURE 3.**
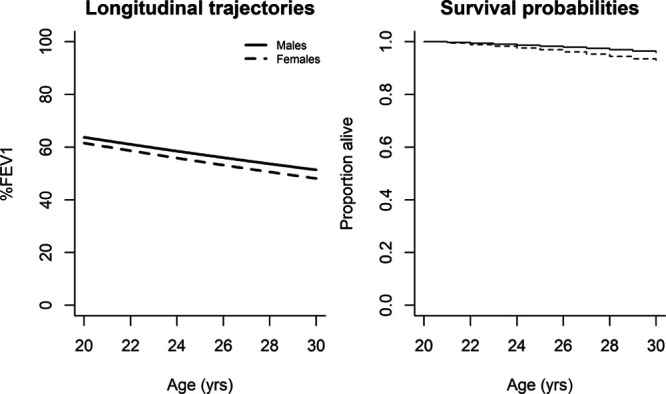
Estimated longitudinal trajectories and survival curves for males and females age 20, born in 1980 with 2 F508 alleles. Effect of sex on forced expiratory volume in 1 second as a percentage of predicted (%FEV1) and survival in the final joint model.

Figure 3 visualizes the association of sex with lung function and survival in the joint model. Males have a more favorable %FEV1 and survival trajectory. Note that the estimated sex effects in Figure 3 are population-averaged effects; that is, they describe average values of %FEV1 for subpopulations of individuals sharing the same explanatory characteristics, rather than for any one individual.

### Association Between Female Sex and Survival Adjusted for Lung Function

In Table 3, we compare the results of four possible survival models that adjust for %FEV1 in various ways. Contrasting the association of sex with survival across these models, in all three of the standard survival models there is a strong association between sex and survival in models without adjustment for %FEV1, and with adjustment for % FEV1 either as a baseline or as a time-varying covariate (effect sizes 0.15, 0.13, 0.14, i.e. males have better survival than females). By contrast, we observe less association between sex and survival after adjusting for %FEV1 (level and rate of decline) in the joint model, with an effect size attenuated by 37% to 0.09 (95% CI = 0.06, 0.12). Table 3 also shows that the genotype effect is reversed once we adjust for longitudinal lung function in the joint model. Residual diagnostics did not raise any concerns about model fit (eAppendix2; http://links.lww.com/EDE/B714).

## DISCUSSION

We apply a novel joint modeling approach, for the first time, to show that about 40% of the association of female sex on survival in cystic fibrosis is explained by the effect of sex on lung function. Both increased rates of decline in lung function, and a decreased overall level of lung function, are associated with an increased risk of death. The strength of this analysis is the use of a joint modeling approach that leads to more robust estimation of both survival estimates and the rate of lung function decline. Our joint modeling approach allows exploration of how aspects of an individual cystic fibrosis patient’s longitudinal profile of %FEV1 are related to their survival prognosis. A key advantage of the joint model is that it estimates the relationship between characteristics of the “true” error-free underlying %FEV1 trace and survival. Our analysis here suggests that measurement error – in conventional approaches to adjusting for %FEV1 in survival analysis – has led to previous estimates of a substantial association between sex and survival. This overestimate was attenuated once error-free %FEV1 was taken into account in the joint model.

Many studies have explored survival in cystic fibrosis,^1,2,13^ and notable among these are the large studies from North America that have used Cox regression to estimate the effect of various covariates on survival chances in registry populations.^5,6,23^ A large number of factors have been identified that may influence survival, with female sex identified most commonly as a risk factor. Other influences include poor respiratory function and risk factors for poor lung function such as *P. aeruginosa* infection status, homozygous delta F508 status, or heterozygous nondelta F508 status, non-white ethnicity, and low income. Many studies have investigated the impact of baseline time-invariant factors on survival, including a recent study using UK data by Keogh and colleagues which showed a clear sex gap, with worse survival for female patients.^13^ Where studies have investigated time varying predictors such as lung function, these have been included in survival models as current values. To our knowledge, our study is one of the first to quantify the association of rate of lung function decline with survival using a joint modeling approach. Another recent study by Keogh et al used landmarking to predict survival in the UK population, and in a two-stage approach used fitted values from a longitudinal mixed-effects model for %FEV1 to estimate current values of %FEV1 for inclusion in the second-stage landmarking analysis.^14^ The study found that current %FEV1 was the strongest predictor of survival. As in our joint model, this two-stage landmarking approach could also be used to estimate the association of the recent rate of decline of %FEV1 with survival.

There is a large literature on lung function decline in people with cystic fibrosis. Konstan et al have undertaken the largest studies to date of %FEV1 in both pediatric and adult cohorts,^24–26^ using a mixed-effects regression approaches, to show that higher baseline %FEV1, *P. aeruginosa* colonization, female sex, and poor nutritional status were amongst the factors associated with a greater decline in lung function in people with cystic fibrosis.^24^ Our analysis contributes to this literature by explicitly quantifying how longitudinal changes in lung function are related to survival. Both an increased rate of decline in lung function, and a decreased overall level of lung function are associated with an increased risk of death in people with cystic fibrosis.

Large national cystic fibrosis patient registry data in different countries have shown that survival for cystic fibrosis females was less than that for matched cystic fibrosis males, in an apparent sex gap. The etiology of this gap is poorly understood, with increased mortality in females seen even after correcting for lung function, suggesting the explanation is likely to be multifactorial. Our analysis adds to this literature, estimating that about 40% of the effect of sex on survival is explained by the impact of sex on lung function decline, which in turn influences survival. There are known effects of sex on lung function and acquisition of *P. aeruginosa* that we have previously identified in this dataset.^12^ Others have suggested alternative biologic reasons, including the impact of estrogen and increased occurrence of cystic fibrosis-related diabetes (CFRD).^3,8^ Social explanations proposed may relate to gender roles, such as a possible propensity to less exercise in childhood in girls, and an increased tolerance of poor nutritional status in adolescent girls with cystic fibrosis, fuelled by the societal pressure to appear thin.^27^

Our analysis suggests that the sex gap may be partly explained by worse lung function in females. We suggest that standard approaches for adjusting for lung function in survival analysis may lead to insufficient adjustment, which may explain the difference between our results, and other large epidemiologic studies of cystic fibrosis. Because lung function is measured with significant error, the associations with other covariates in the analysis may be confounded by residual effects of lung function. In our joint model, which more precisely adjusts for underlying lung function, differences in lung function explain about 40% of the sex difference in mortality. For instance, one of the largest studies to explore the gender gap in cystic fibrosis was Rosenfeld and colleagues’ analysis of US registry data,^5^ which showed a significant association of sex on survival and lung function. The authors show that pulmonary function was the only risk factor that explained a portion of the observed gender-related difference in survival. Among the subjects 1-20 years of age, females had a hazard ratio of 1.7 in the unadjusted Cox regression analysis, but this was attenuated to 1.5 while adjusting for %FEV1 as a time-varying covariate. The authors suggest that differences in pulmonary function did appear to explain a small portion of the excess female mortality, but no other factor further accounted for the gender gap. A more recent large study of US registry data identified a survival advantage for males compared with females, with a 19% (CI = 13%, 24%) lower adjusted risk for death in males as compared with females,^28^ and the authors further highlight that the reasons for this are not well-understood. However, this analysis did not adjust for lung function at a time-varying covariate but focused on adjustment for baseline factors at the time of diagnosis, which can be used by clinicians at diagnosis to inform discussions about patient prognosis.

To our knowledge, this is the first study to explore the sex effect in cystic fibrosis using joint modeling approaches, though previous studies have used cystic fibrosis data to develop joint modeling methods.^19^ Key strengths of this study include the population-wide coverage of the UK Cystic Fibrosis Registry and the high quality of the data.^20^ Although we have not presented our analysis as formal mediation analysis, the steps we have undertaken map onto the Baron and Kenny^29^ steps for mediation analysis, subject to a number of assumptions (see eAppendix 2; http://links.lww.com/EDE/B714). Newer methods for mediation analysis based on the counterfactual framework, and software to implement them, are developing rapidly, and further work is warranted to understand how joint models can be used within the potential outcomes framework. Such approaches could be usefully applied to range of mediation questions in cystic fibrosis epidemiology. For example, while not the main focus of our analysis here, the reversal of the genotype effect in our joint model suggests that heterozygotes for del508, compared with those with one or no del508 alleles, have better survival after adjustment for lung function. Further analyses could explore decomposing the sex effect on survival through a potential effect of sex on *P. aeruginosa* acquisition which is known to impact lung function decline.

There are a number of limitations to our analysis: First, it relies on retrospective, routinely collected data. Second, our joint modeling approach assumes that %FEV1 measurements are independent of survival given an individual’s random-effect values, which may not be appropriate. Third, we did not explore the full range of mediating pathways through which sex may impact survival. Our main hypothesis related to the sex term in the survival submodel, and we did not seek to adjust for other potential downstream mediators of the association between sex and survival in the survival submodel, such as CFRD and infection status indicators, instead adjusting for slope and overall level of lung function as captured in our longitudinal submodel. Whilst the random effects included in the longitudinal component of our joint model capture residual underlying baseline heterogeneity they are unlikely to adequately capture time-varying effects such as infections. The impact of infection on survival, however, is likely to be largely mediated through impacts on lung function. A recent study has illustrated how left truncation can lead to biased estimates in the context of joint models.^30^ We aimed to limit the impact of left truncation by including the birth year as a covariate in the longitudinal and survival submodels. The effect of birth year here is driven by both cohort effects and left truncation. Another limitation of our study is that it is debatable how transplanted individuals should be handled in a joint model.^14^ For the purposes of this analysis, we included post-transplant FEV1 measures and deaths, but further work is needed to understand how best to take account of transplantation and post-transplant survival in the context of a joint modeling analysis.

In summary, we have applied the Barrett et al^19^ approach to joint modeling in cystic fibrosis to address the question of how the sex effect on survival is explained by lung function. Our analysis suggests that if the lung function gap between males and females can be narrowed, this should also narrow the survival gap. Our analysis approach can be applied to similar etiologic questions in longitudinal Cystic Fibrosis Registry data and can be used to more accurately adjust for time-varying covariates measured with error, such as lung function, in survival analyses in cystic fibrosis and other conditions.

## ACKNOWLEDGMENTS

We thank the UK Cystic Fibrosis Trust for access to the UK Cystic Fibrosis Registry and all the Centre Directors for the input of data to the registry. Elaine Gunn assisted with access to the data. JKB was supported by the MRC (grant number G0902100 and unit programme number MC_UU_00002/5). DTR is funded by the MRC on a Clinician Scientist Fellowship (MR/P008577/1). The work was supported by the Strategic Research Centre “EpiNet: Harnessing data to improve lives” funded by the Cystic Fibrosis Trust.

## Supplementary Material


