# Supplementary material for: Explaining the Sex Effect on Survival in Cystic Fibrosis: a Joint Modeling Study of UK Registry Data
Source: Epidemiology. 2020 Aug 6;31(6):872–9. doi: 10.1097/EDE.0000000000001248 (PMC7523568; doi:10.1097/EDE.0000000000001248)
Supplement: Supplementary file 1 [file ede-31-872-s001.docx]

**eAppendix 1. Description of the joint model**

Repeated %FEV_1_ measurements and survival data were modelled using a joint model with shared random effects between the measurement sub-model and the survival sub-model (for an introduction to joint models see Asar *et al*, 2015). If $f(y)$ denotes the distribution of the measurement process and $f(t)$ denotes the distribution of the survival process, then a key assumption of joint models is that the measurement and survival processes are independent conditional on the random effects $u$, i.e. the joint likelihood factorises as

$f\left( y,t,u \right)=f\left( y | u \right)f\left( t | u \right)f\left( u \right) .$ (1)

The joint model was fitted by maximising the likelihood (1), thus all repeated measurement and survival parameters are estimated simultaneously and random effects are predicted by their expectations conditional on both repeated measurements and survival data.

# Model specification

Let $m$ denote the number of patients, and $n_{i}$ the number of follow-up measurements on the *i*th patient. Let $x_{i}$ denote the vector of baseline covariates (birth year, sex and coded F508 allele status), $a_{ij}$ the age, and $Y_{ij}$ the measured value of %FEV_1_, for the *i*th patient at their *j*th follow-up time $t_{ij}$. Let $w_{ij}$ denote the vector of interactions ($\text{birth year}\times\text{sex}$, $\text{birth year}\times\text{F508}$, $a_{ij}\times\text{sex}$, $a_{ij}\times\text{F508}$). Let $(U_{i},V_{i})$ be independent and identically distributed bivariate Normal random effects with means zero, variances $\sigma_{U}^{2}$ and $\sigma_{V}^{2}$ , and correlation $\rho$. Finally, let $Z_{ij}$ be independent and identically distributed Normal random variables with mean zero and variance $\tau^{2}$.

*U*

*V*

Our measurement sub-model takes the form

$\begin{aligned} Y_{ij}&=x_{i}^{T}\beta_{0}+a_{ij}\beta_{1}+a_{ij}^{2}\beta_{2}+w_{ij}^{T}\beta_{3}+U_{i}+V_{i}a_{ij}+Z_{ij} : j=1,\ldots,n_{i}; i=1,\ldots,m \\ &=\mu_{i}\left( a_{ij} \right)+Z_{ij} \end{aligned}$ (2)

where $\mu_{i}\left( a \right)$ is the underlying error-free value of the $i$th patient’s %FEV_1_ at age $a$.

Our survival sub-model partitions the follow-up period for each subject into discrete, one-year intervals and assumes that the probability of survival in the $k$th such interval is

$p_{ik}=\Phi\left[ x_{i}^{T}\alpha_{0}+a_{ik}\alpha_{1}+\gamma_{0}\mu_{i}\left( a_{ik} \right)+\gamma_{1}\mu_{i}^{'} \right] ,$ (3)

where $\Phi[\cdot]$ denotes the standard Normal distribution function, $x_{i}$ is again the vector of baseline covariates (birth year, sex and coded F508 allele status), $a_{ik}$ is the age of the $i$th patient at the mid-point of the $k$th follow-up interval and $\mu_{i}'$ is the rate of change in the $i$th patient’s underlying error-free %FEV_1_.

Conditional on the random effects $(U_{i},V_{i})$ for the $i$th patient, the probability that they deliver

$n_{i}$ measurements is

$$P_{i}\left( U_{i}, V_{i} \right)= \left\{ \sum_{k=1}^{n_{i}} p_{ik} \right\}\times\left( 1-p_{n_{i}+1} \right)^{c_{i}}$$

where $c_{i}=1$ if the patient in question dies between the scheduled times of their $n_{i}$th and $(n_{i}+1)$st measurements and $c_{i}=0$ otherwise. Also conditional on $(U_{i},V_{i})$, the joint distribution of

the $i$th patient’s measurement sequence $Y_{i}=(Y_{i1},\ldots,Y_{in_{i}})$ is multivariate Normal with mean vector $M_{i}\left( U_{i},V_{i} \right)=(\mu\left( a_{i1} \right),\ldots,\mu(a_{in_{i}}))$ and variance matrix $\tau^{2}I$, where $I$ is the identity matrix.

*i*

The contribution of the $i$th patient to the likelihood function for the data follows by combining

(2) with (3) and marginalising with respect to the bivariate Normal distribution of $(U_{i},V_{i})$, to give

$$\begin{aligned} L_{i}=\int\int P_{i}\left( U,V \right)\times\text{MVN}_{n_{i}}\left( Y_{i};M_{i}\left( U,V \right),\tau^{2}I \right)\times\text{MVN}_{2}\left( \left( U,V \right);0,R \right)dUdV .\#\left( 4 \right) \end{aligned}$$

In (4), *R* is the symmetric 2 by 2 matrix with diagonal elements $\sigma_{U}^{2}$ and $\sigma_{V}^{2}$, and off-diagonal

elements $\sigma_{U}\sigma_{V}\rho$, and $\text{MVN}_{d}(Y,M,\Sigma)$ denotes the probability density of a multivariate Normal

distribution with mean vector $M$, variance matrix $\Sigma$ and argument $Y$ (Barrett *et al*, 2015). The log-likelihood is then given by the summing the logarithm of (4) over patients, hence

$$\mathcal{l}\left( \theta\right)=\sum_{i=1}^{m} \text{log} L_{i} ,$$

where *θ* denotes the complete set of model parameters.

To fit the model, we estimate $\theta$ by numerical maximisation of $\mathcal{l}\left( \theta\right)$, using the R function optim with the following argument settings: method="BFGS"; reltol=1e-9; maxiter=10000. We then use the R function hessian from the numDeriv package to obtain the numerical approximation to the Hessian matrix, from which we calculate standard errors and report 95% confidence intervals as estimates *±*2 standard errors.

# Calculation of hazard ratios

To express covariate effects as hazard ratios, we approximate the hazard ratio by the risk ratio of experiencing an event in the next year

$$\text{HR}\approx\frac{1-\hat{p}_{i_{2}}}{1-\hat{p}_{i_{1}}}$$

where $\hat{p}_{i_{1}}$ is the estimated risk given by equation (3) using parameter estimates from the fitted model for patient 1 with covariates $x_{i_{1}}$ and $a_{i_{1}}$, and random effects $U_{i_{1}}$ and $V_{i_{1}}$. And similarly for $\hat{p}_{i_{2}}$.

We estimate the variance for the log HR using the delta method

$$V\left( \text{log} \text{HR} \right)\approx\left( \begin{matrix} \frac{\partial\text{log} \text{HR}}{\partial\eta_{i_{1}}} & \frac{\partial\text{log} \text{HR}}{\partial\eta_{i_{2}}} \end{matrix} \right)\left( \begin{matrix} V\left( \eta_{i_{1}} \right) & \text{Cov}\left( \eta_{i_{1}},\eta_{i_{2}} \right) \\ \text{Cov}\left( \eta_{i_{1}},\eta_{i_{2}} \right) & V\left( \eta_{i_{2}} \right) \end{matrix} \right)\left( \begin{matrix} \frac{\partial\text{log} \text{HR}}{\partial\eta_{i_{1}}} \\ \frac{\partial\text{log} \text{HR}}{\partial\eta_{i_{2}}} \end{matrix} \right)$$

where $\eta_{i_{1}}$ and $\eta_{i_{2}}$ are the linear predictors for the probit survival model for patients 1 and 2 respectively, and

$$\frac{\partial\text{log HR}}{\partial\eta_{i_{1}}}=\frac{\phi\left[ \eta_{i_{1}} \right]}{1-\Phi\left[ \eta_{i_{1}} \right]} , \frac{\partial\text{log HR}}{\partial\eta_{i_{2}}}=\frac{-\phi\left[ \eta_{i_{1}} \right]}{1-\Phi\left[ \eta_{i_{2}} \right]}$$

and $\phi[\cdot]$ denotes the standard Normal density function. The variance and covariance of the

linear predictors is estimated as, e.g. for $\eta_{i_{1}}$

$$V\left( \eta_{i_{1}} \right)=\left( \begin{matrix} x_{i_{1}}^{T} & a_{i_{1}} & \mu_{i_{1}} & \mu_{i_{1}}^{'} \end{matrix} \right) \Sigma\left( \begin{matrix} x_{i_{1}}^{T} \\ a_{i_{1}} \\ \mu_{i_{1}} \\ \mu_{i_{1}}^{'} \end{matrix} \right)$$

where $\Sigma$ is the variance matrix of the vector of parameter estimates $\left( \hat{\alpha}_{0},\hat{\alpha}_{1},\hat{\gamma}_{0},\hat{\gamma}_{1} \right)^{T}$ , and is estimated by the inverse of the Fisher information matrix. Confidence intervals are then estimated on the log HR scale, and exponentiated to the HR scale.

# References

Asar, O¨ ., Ritchie, J., Kalra, P.A. and Diggle, P.J. (2015). Joint modelling of repeated measure- ment and time-to-event data: an introductory tutorial. *International Journal of Epidemiology*, **44**, 334–344.

Barrett, J., Diggle, P.J., Henderson, R. and Taylor-Robinson, D. (2015). Joint modelling of repeated measurements and time-to-event outcomes: flexible model specification and exact likelihood inference. *Journal of the Royal Statistical Society*, B **77**, 131–148.

**eAppendix 2. Description of the joint model Exploratory plots and model checks**

**Histogram of age at entry to cohort**

**
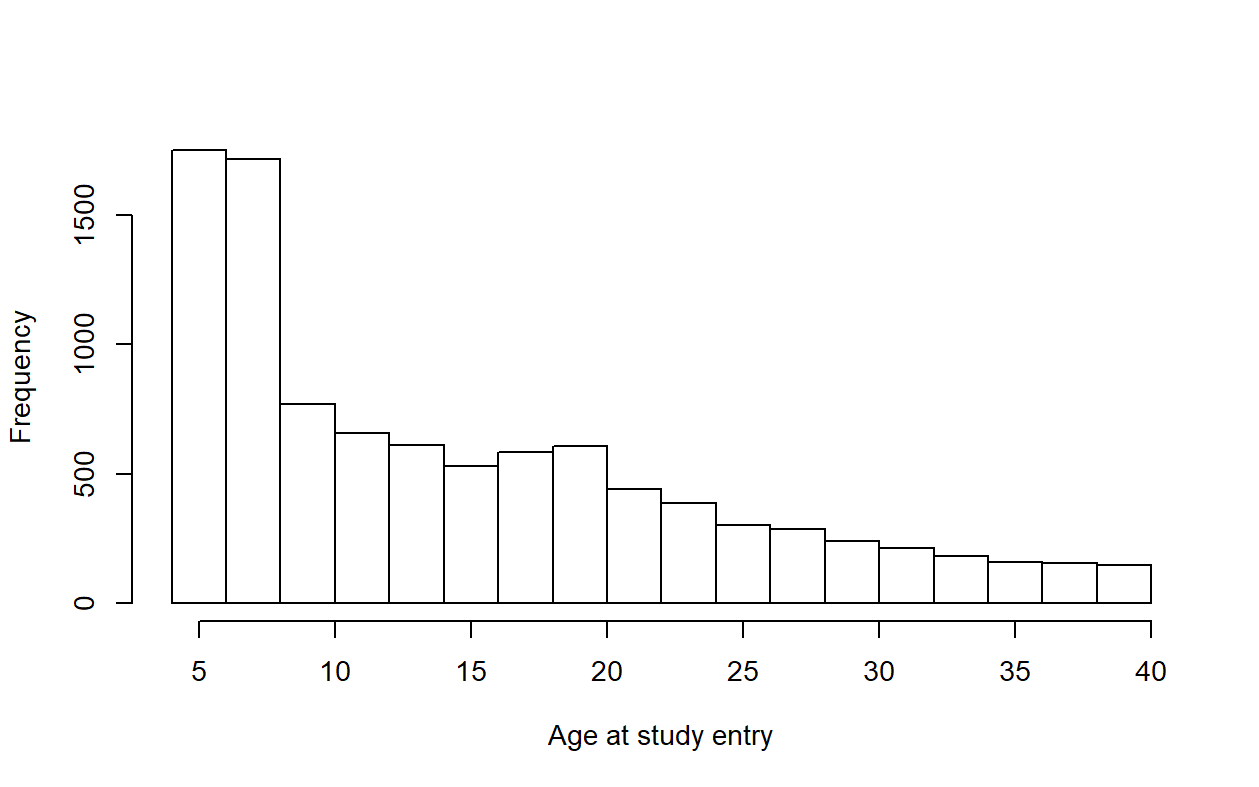
**

**Supplementary Figure 1:** Histogram of age at study entry.

|  | **Female** | **Male** | **All** |
| --- | --- | --- | --- |
| Number post-transplant (of any type) at baseline | 62 | 91 | 153 |
| Number receiving lung transplant post-baseline | 215 | 197 | 412 |
| Number of deaths amongst those receiving  lung transplant post-baseline | 68 | 47 | 115 |

**Supplementary Table 1:** Numbers of transplants by sex

**Random effect parameter estimates for joint model**

|  | Estimate (95% CI) |
| --- | --- |
| Random intercept S.E. | 22.24 (21.78, 22.71) |
| Random slope S.E. | 1.47 (1.44, 1.50) |
| Correlation between random intercept and random slope | -0.56 (-0.57, -0.54) |
| Residual variance in %FEV_1_ model | 9.23 (9.18, 9.29) |

**Supplementary Table 2:** Joint model parameter estimates and 95% CIs for all parameters not reported in Tables 2 and 3.

**Spaghetti plots**

**
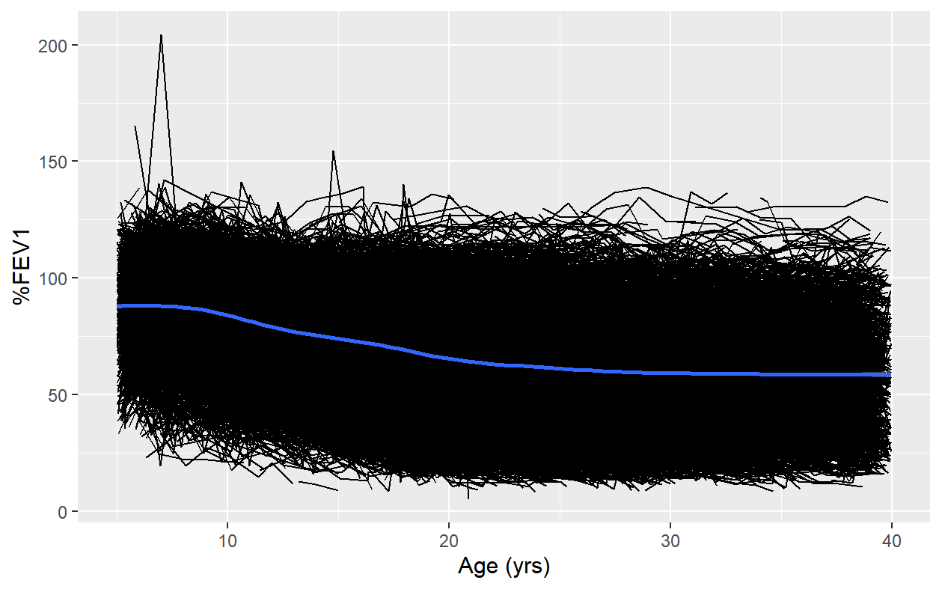

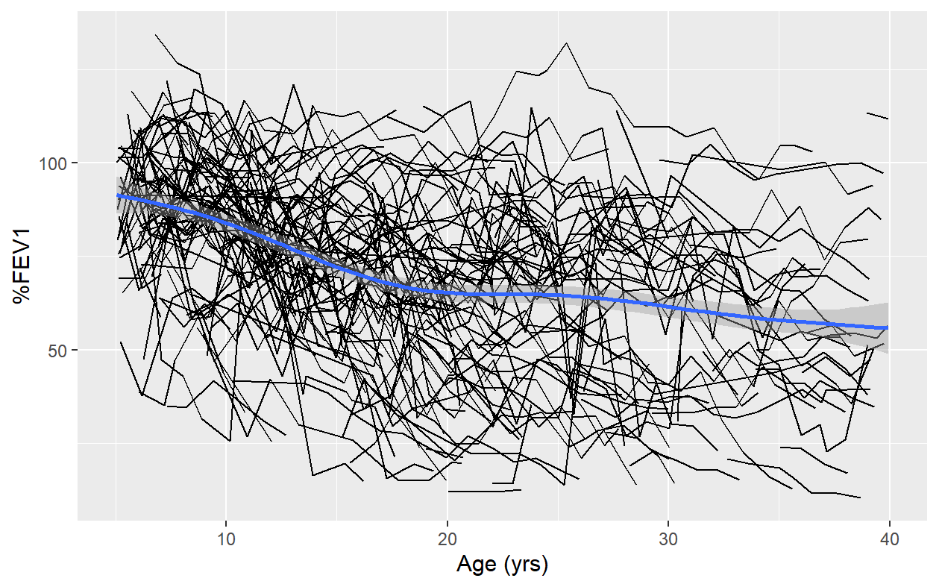
**

**Supplementary Figure 2:** Spaghetti plots with loess smoothers for the full longitudinal data sample (left) and a random sample of 200 individuals (right)

**Residual plots**

**
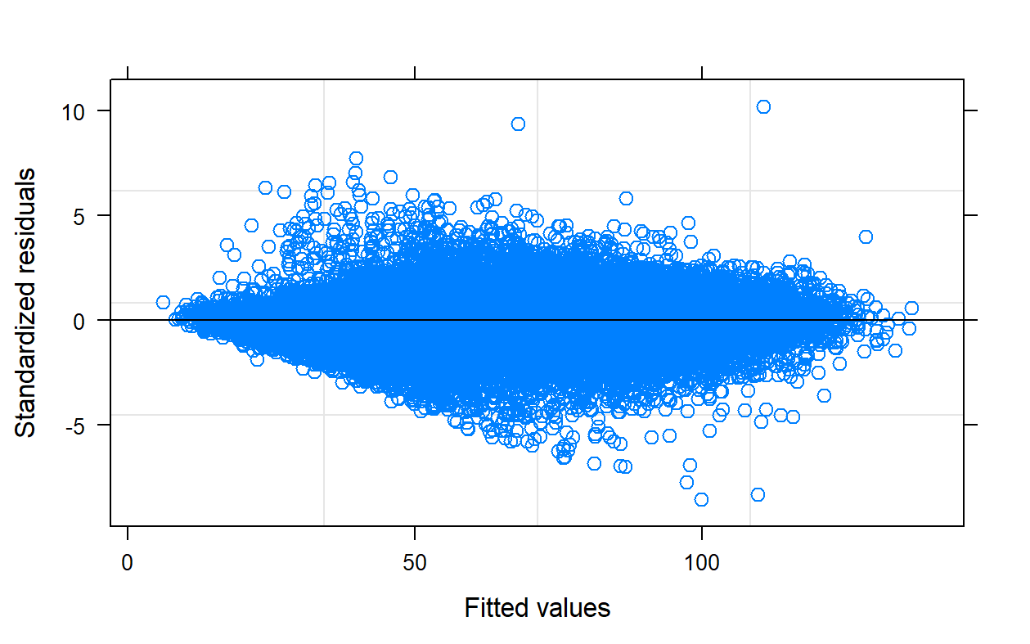

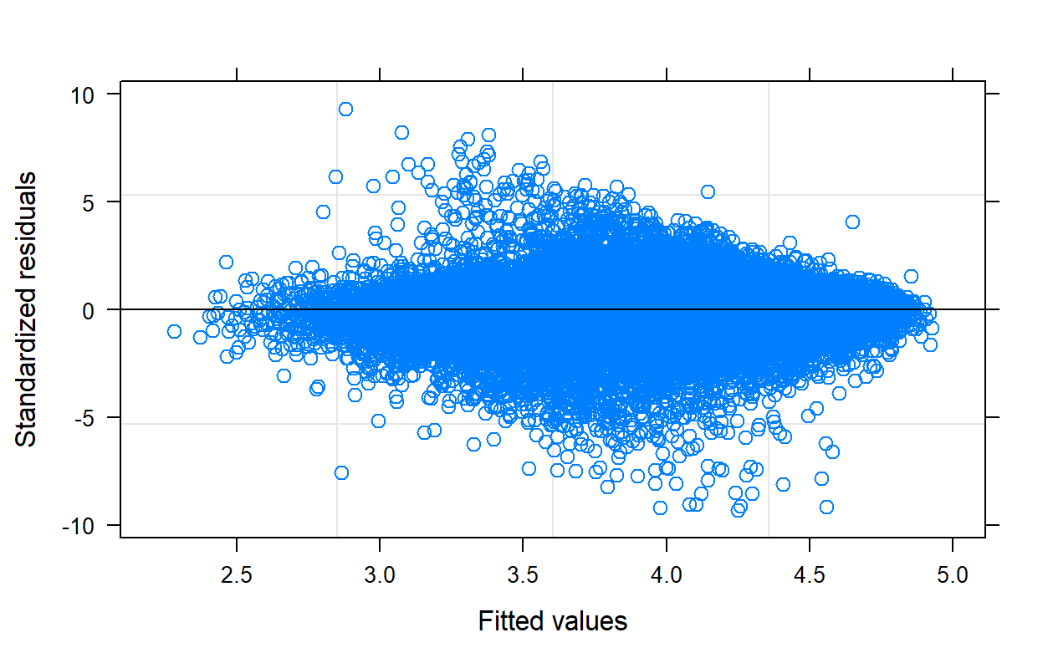
**

**Supplementary Figure 3:** Residual diagnostic plot for longitudinal model - %FEV1 outcome (left) and log%FEV1 (right).

**Empirical variogram**


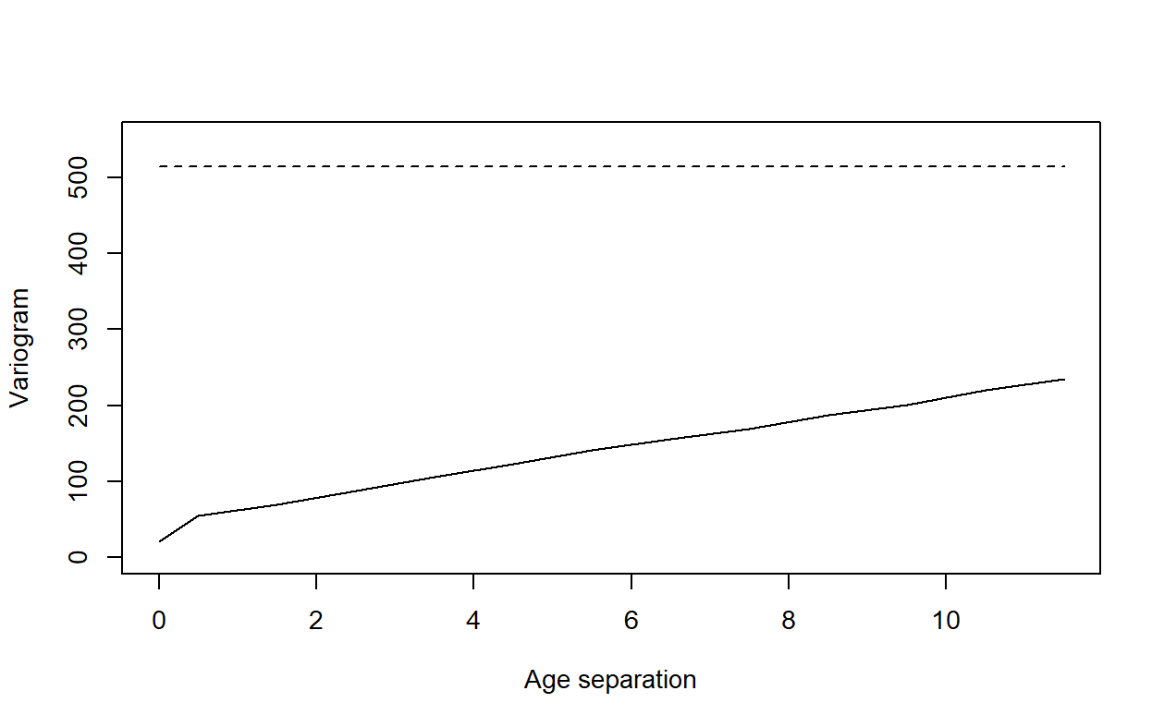


**Supplementary Figure 4:** Empirical variogram for observed %FEV1 measurements. The dotted line represents the estimated total variance.


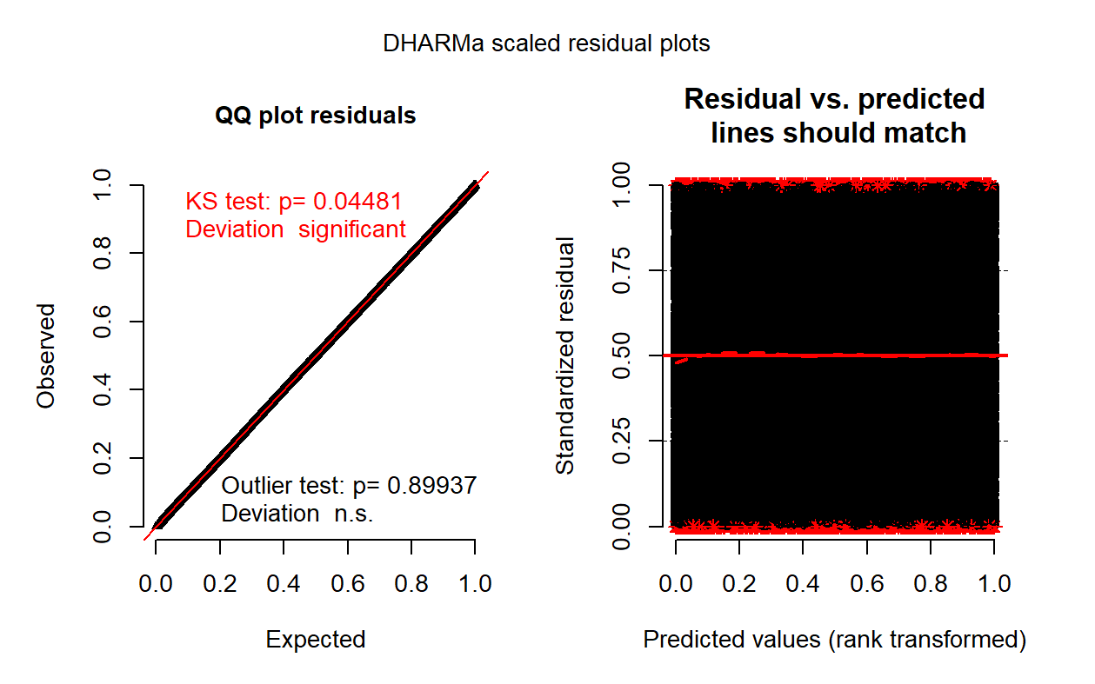


**Supplementary Figure 5:** Residual diagnostic plots for survival model. For the probit model residual plots were created using the DHARMa package in R

**eAppendix 3. Testing for mediation**

The figure below shows the directed acyclic graph (DAG) for a causal mediation analysis.

**
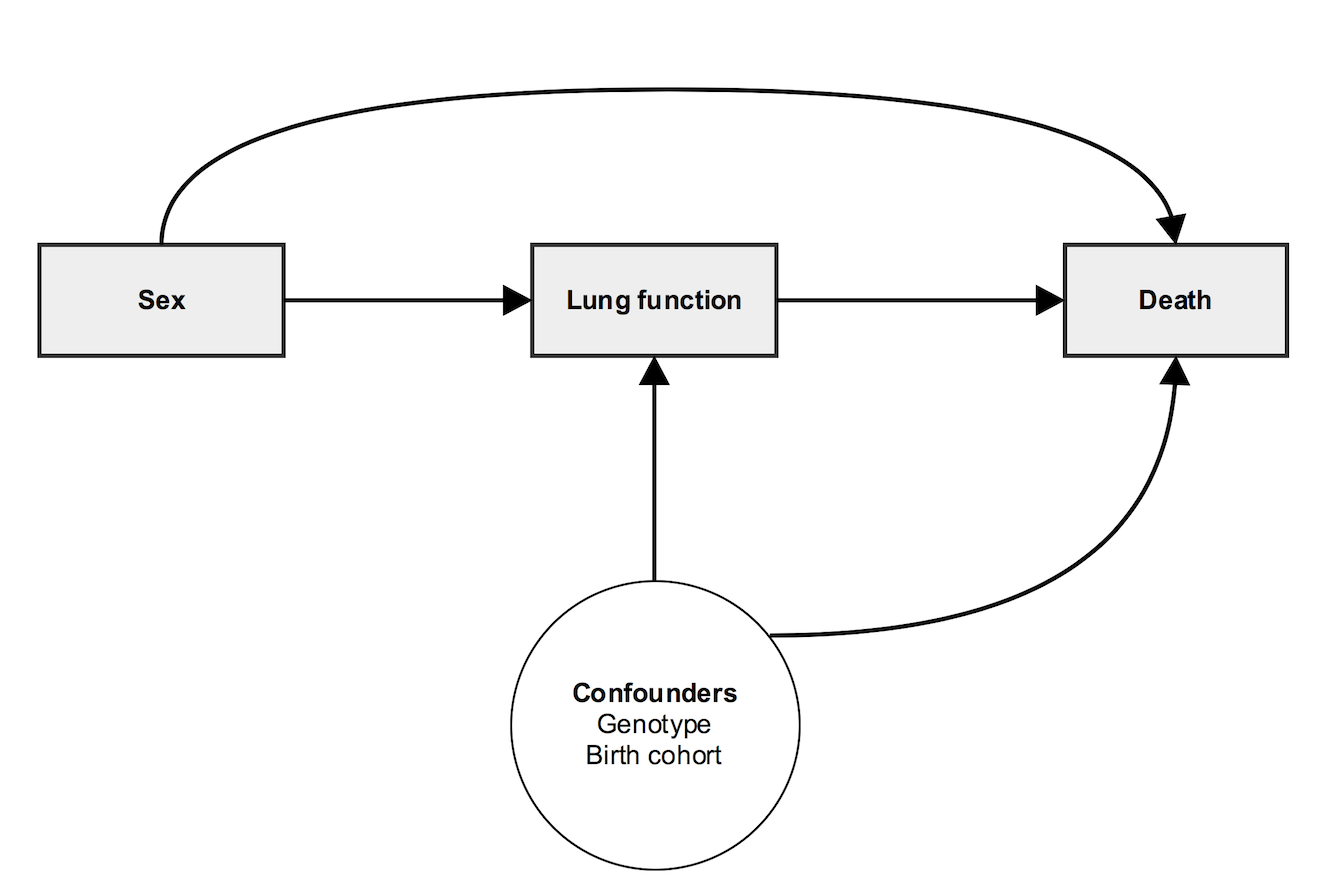
**

Baron and Kenny outline four steps in establishing mediation(1):

Step 1:  Show that the causal variable (sex) is correlated with the outcome (death).  This is demonstrated in the probit survival models (table 3), whereby female sex is associated with increased risk of death.

Step 2: Show that the causal variable is correlated with the mediator.  This is demonstrated in table 2 (the FEV1 submodel) whereby female sex is associated with an increased rate of decline of %FEV1.

Step 3:  Show that the mediator affects the outcome variable, whilst controlling for the causal variable. This is demonstrated in the probit survival models and the joint survival model in table 3 whereby worse lung function is associated with increased risk of death, whilst adjusting for sex.

Step 4:  To establish that M completely mediates the exposure-outcome relationship, the effect of exposure on outcome controlling for the mediator should be zero. This is demonstrated in our joint model where we more accurately adjust for lung function, and the lower confidence bound for the sex effect is essentially zero; but not in the probit model.

**Limitations:**

Standard approaches to using survival models for mediation by adding a mediator as a covariate to the hazard model and then seeing if the coefficient for the exposure changes is subject to some important limitations. These include the no-confounding assumptions outlined below; and also a number of modelling assumptions, because the hazards ratio is not in general a ‘collapsible’ measure. However, the latter has been shown not to be problematic when the outcome is relatively rare (13% mortality in our study).(2)

The no-confounding assumptions mean that four types of confounding are adjusted for: (1) confounding of the exposure-outcome relationship; (2) confounding of the mediator-outcome relationship; (3) confounding of the exposure-mediator association; and (4) mediator-outcome confounders also affected by the exposure.

Alternative methods for mediation analysis are continuously being developed, but have their limitations in terms of application in routinely available software, and to our knowledge these have not been routinely applied in the context of joint models.(3)

1. Mediation (David A. Kenny) [Internet]. [cited 2018 Jul 20]. Available from: http://davidakenny.net/cm/mediate.htm

2. VanderWeele TJ. Unmeasured confounding and hazard scales: sensitivity analysis for total, direct, and indirect effects. Eur J Epidemiol. 2013 Feb;28(2):113–7.

3. Richiardi L, Bellocco R, Zugna D. Mediation analysis in epidemiology: methods, interpretation and bias. Int J Epidemiol. 2013 Sep 9;dyt127.
